# Evaluation of patient doses for routine digital radiography procedures toward establishing an institutional diagnostic reference levels: A case study in Sri Lanka

**DOI:** 10.1002/acm2.13852

**Published:** 2022-11-30

**Authors:** Sachith Welarathna, Sivakumar Velautham, Mihira Wanninayake, Sivananthan Sarasanandarajah

**Affiliations:** ^1^ Postgraduate Institute of Science University of Peradeniya Peradeniya Sri Lanka; ^2^ Horizon Campus Malabe Sri Lanka; ^3^ Department of Physics University of Peradeniya Peradeniya Sri Lanka; ^4^ School of Health Science British College of Applied Studies Colombo Sri Lanka; ^5^ Peter MacCallum Cancer Institute and RMIT University Melbourne Victoria Australia

**Keywords:** diagnostic reference levels, dose optimization, kerma‐area product, patient dosimetry, radiation protection

## Abstract

The present study was conducted as part of a comprehensive work to establish National Diagnostic Reference Levels (NDRLs) in Sri Lanka for the first time. DRLs can be used as an effective optimization tool for identifying unusually high or low patient doses during X‐ray examinations. This study aims to propose institutional DRLs (IDRLs) by measuring the kerma‐area product (KAP) of adult patients undergoing routine projection X‐ray examinations. The median and the 75th percentile KAP values obtained were compared with that of the single institution KAP values reported from India and Greece. This descriptive cross‐sectional study was conducted in a public hospital in Uva province, Sri Lanka, with 400 adult patients aged 18–87 years and weighing 58 ± 20 kg. The patient‐specific information (age, sex, weight, and height) and corresponding exposure parameters (tube voltage and current‐exposure time product) were obtained. The KAP values were measured, and descriptive statistics were utilized for data analysis. The median KAP values obtained were proposed as IDRLs. The IDRLs in Gy.cm^2^ were 0.23 for cervical spine anterior–posterior (AP), 0.19 for cervical spine lateral (LAT), 0.10 for chest posterior–anterior (PA), 0.06 for knee joint AP, 0.05 for knee joint LAT, 1.47 for KUB AP, 0.85 for lumbar spine AP, 1.97 for lumbar spine LAT, 0.29 for shoulder joint AP, 0.61 for skull PA, and 0.60 for skull LAT examinations. The maximum to minimum ratio of KAP values ranged from 2.4 for KUB AP to 6.3 for the cervical spine AP examinations. The median and the 75th percentile of most of the examinations were comparable to corresponding KAP values reported by the countries mentioned above, except for the skull PA and LAT examinations. Accordingly, interquartile ranges of exposure parameters are recommended for skull examinations to improve the optimization of patient doses.

## 1 INTRODUCTION

Over the past decades, the use of ionizing radiation in medicine has expanded rapidly worldwide. It is identified that medical exposures in diagnostic radiology procedures contribute significantly to artificial sources of radiation.^1^ A report published by the United Nations Scientific Committee on the Effects of Atomic Radiation (UNSCEAR) estimates that over 2.6 billion conventional radiology examinations (including projection radiography and fluoroscopy) were performed annually worldwide between 2009 and 2018.^2^ This accounts for 62.6% of the annual number of medical radiological examinations and contributes to 23.0% of the annual collective effective dose.^2^ Apart from the enormous benefits of X‐ray examinations, the patients are exposed to substantial radiation doses, which may cause stochastic and deterministic effects (tissue reactions) that could be harmful to the patients.^3^ Therefore, patient doses associated with X‐ray examinations must be assessed, justified, and optimized in terms of benefits and risks to improve patient protection. According to the International Commission on Radiological Protection (ICRP), there are two basic principles of radiation protection implemented in medical exposures of patients: optimization and justification.^4^ The purpose of optimization and justification is to keep the patient doses as low as reasonably achievable (ALARA) without compromising intended diagnostic information.^5^

The concept of diagnostic reference levels (DRLs) was introduced by ICRP as an effective optimization tool for identifying unusually high or low patient doses during radiographic procedures, as both may have adverse effects on patients.^5^ DRLs are considered as indicators that can be used to optimize patient protection by identifying unusually high or low patient doses that are not justified by image quality requirements.^6^ In addition to the ICRP, many professional and regulatory organizations, including the International Atomic Energy Agency, the European Commission, the American College of Radiology, the American Association of Physicists in Medicine, and the United Kingdom Health Security Agency (formerly named as Public Health England), recommend the use of DRLs in medical exposures.^4^, ^7^, ^8^, ^9^, ^10^

It is important to note that the DRLs do not represent a demarcation between good and bad medical practices.^5^ Instead, they can promote optimum dose ranges for X‐ray examinations, provide a common dose metric for comparison of patient doses, and minimize dose variations in standard radiographic procedures.^11^, ^12^ The entrance surface air‐kerma and kerma‐area product (KAP) are used as easily measurable and practicable dose quantities to set DRLs in projection radiographic examinations.^5^, ^13^ The KAP, which is also known as the dose‐area product (DAP), is the product of incident air‐kerma (Gy) and the corresponding irradiated area (cm^2^) and is typically expressed in Gy.cm^2^.^14^ The ICRP publication 135 recommends the 75th percentile of the median KAP distributions of healthcare facilities in a country can be used to establish the national DRLs (NDRLs).^5^ Besides, the median (50th percentile) of KAP distributions (also known as typical values) obtained for routine X‐ray examinations in a specific healthcare facility can be adopted as facility reference levels or institutional DRLs (IDRLs) to further optimize patient doses by comparing them with local, national, or regional dose levels.^5^, ^12^, ^15^ Generally, IDRLs for standard X‐ray examinations should not exceed local DRLs (LDRLs) or NDRLs during good and normal practices unless clinically justified; however, if IDRLs are consistently exceeded, appropriate corrective measures should be taken to avoid unnecessary radiation risks.^11^ This could involve changes in the radiographic techniques employed and/or the radiographic equipment.

Furthermore, dosimetric surveys conducted in certain countries revealed that there are wide variations in patient doses for the same X‐ray examination even within the same institution.^16^, ^17^ These variations are mainly attributed to differences in patient size, clinical condition, competencies and experience of radiologic technologists, equipment conditions, and radiographic techniques utilized.^17^, ^18^ However, such variations should be minimized or prevented in good and normal practices, and further actions should be taken to optimize the doses. Therefore, DRLs are usually specified for standard‐sized patients or phantoms for a specific X‐ray examination but not for individual patients.^5^ It was found that the radiation doses associated with X‐ray examinations are significantly dependent on patient size.^15^, ^19^ According to the ICRP publication 135, standardization of patient size is usually achieved through the selection of a representative sample of adult patients with respect to mean population weight and within a certain range.^5^ However, the mean weight of a population demographically varies due to differences in patients’ anthropometric measurements. According to the World Health Organization (WHO) report, the mean weight of an adult patient in Sri Lanka is 58 kg.^20^

Although various dosimetry quantities are available, the KAP is considered as one of the most appropriate dosimetry quantities. It has increasingly been used in many countries to estimate organ doses, effective doses, and total energy deposited in the patient and thus better express the biological effects (stochastic effects) associated with X‐ray examinations.^14^ In addition, the KAP is a more useful dose descriptor to assess machine performance and quality assurance.^21^ The DRL process should be reviewed and revised periodically when substantial technological advances become available.^5^ Over the past years, the LDRLs and NDRLs have been established in many countries, serving as a guide for reducing unnecessary patient doses and improving radiology practice. For example, in Europe, 81% of countries have introduced DRLs for projection radiographic examinations.^22^ Although a considerable number of X‐ray examinations are performed annually in Sri Lanka, only a few dosimetric studies have been published for projection radiography, and DRLs are yet to be established. Therefore, the present study aims to measure the KAP of adult patients undergoing routine projection X‐ray examinations to propose IDRLs and compare the results of similar studies conducted in India and Greece for further dose optimization.

## 2 MATERIALS AND METHODS

This study was performed in an X‐ray facility of a well‐established radiology department of a public hospital in Uva province, Sri Lanka. The data collection was performed from January 2022 to May 2022 without affecting the routine workflow in the hospital and without any additional radiation dose to the patients. Ethical approval for this study was obtained from the hospital ethical review committee.

### 2.1 Patient groups

The study group consisted of 400 standard‐sized adult patients who were aged 18 years or older. The weight criterion of 58 ± 20 kg (38–78 kg) was used in this study to select the standard‐size patients from the initial sample of 480 patients. Informed consent was obtained from all participants before initiating X‐ray examinations. Pregnant women and critically ill patients were excluded. Patient‐specific information such as sex, age, weight, and height were obtained for each patient prior to the examination.

### 2.2 X‐ray examinations

The following routinely performed projection X‐ray examinations, namely cervical spine anterior–posterior (AP), cervical spine lateral (LAT), chest posterior‐anterior (PA), knee joint AP, knee joint LAT, KUB (kidney, ureter, and bladder) AP, lumbar spine AP, lumbar spine LAT, shoulder joint AP, skull AP, and skull LAT were investigated. The cervical spine AP, cervical spine LAT, chest PA, shoulder joint AP, skull AP, and skull LAT examinations were acquired in the erect position, while other examinations were taken in the supine position. The AP, PA, and LAT examinations of the same patient were considered separate examinations to estimate the patient doses.

### 2.3 X‐ray system and KAP meter

All the examinations were performed on a digital radiography (DR) system equipped with flat‐panel detector, which was manufactured by Shimadzu Corporation (model RADspeed Pro UD150L‐40E), Japan. The X‐ray system was equipped with 2.5 mm Aluminum (Al) of total filtration, including 1.0 mm Al from the X‐ray tube (type ‐ 0.6/1.2P13DK‐85) and 1.5 mm Al from the X‐ray collimator (type ‐ R‐20J). The X‐ray system employed in this study was operated entirely in a manual exposure control (MEC) mode. Therefore, examination‐specific radiographic exposure parameters such as tube voltage (kVp) and tube current‐exposure time product (mAs) were also obtained from the console of the X‐ray unit for each patient. The KAP values were directly measured by using a KAP meter and expressed in Gy.cm^2^ (VacuDAP Bluetooth, manufactured by VacuTec Meßtechnik GmbH, Germany, with a resolution of 0.01 μGy.m^2^ and dimensions of an active area of 147 × 147 mm^2^). The KAP meter used in this study was a factory‐calibrated device at 70 kV with a tube filtration of 2.5 mm Al without additional absorbers. Also, it had a diagnostic energy range of 40–150 kVp. The KAP meter consisted of two main parts, and the transparent ionization chamber with integrated electronics was installed perpendicular to the central beam axis under the exit window of the X‐ray collimator without interfering with the X‐ray field.

### 2.4 Statistical analysis—determination of IDRLs

The obtained patient‐specific information, KAP measurements, and exposure parameters were reviewed, organized, and classified based on the examination type. The statistical analysis was performed by using IBM SPSS Statistics Version 26.0 (Statistical Package for the Social Sciences; IBM Corp., Armonk, NY, USA) and Microsoft Excel 365 (Microsoft Corp., Redmond, Washington, USA). The Shapiro‐Wilk test and Z‐test were used to check the normality of the KAP distribution (*p* < 0.05). The descriptive statistics of KAP (Gy.cm^2^), mean, median, standard deviation (SD), range, 25th percentile, 75th percentile, and maximum to minimum ratios were calculated and illustrated throughout the box plots for each X‐ray examination.

## 3 RESULTS

This study included a total of 400 standard‐sized adult patients, including 56.2% males and 43.8% females, who underwent 11 types of projection X‐ray examinations. Table 1 summarizes the frequencies of X‐ray examinations and the mean and range of patient‐specific information such as age, weight, height, and body mass index (BMI) for eleven types of projection X‐ray examinations. The mean (range) of age, weight, height, and BMI of the study group were 52.3 (18–87) years, 56.4 (38–78) kg, 1.58 (1.37–1.83) m, and 22.6 (14.3–35.1) kg m^−2^ respectively. It was found that the most frequently performed X‐ray examination was chest PA, which accounts for 27.5% of the total examinations carried out in this hospital.

**TABLE 1:** Descriptive statistics of the patient‐specific information (age, weight, height, and body mass index [BMI])

				Patient‐specific information							
		Number of patients		Age (years)		Weight (kg)		Height (m)		BMI (kg m^−2^)	
Examination type	Number of exposures(n)	Male	Female	Mean	Range	Mean	Range	Mean	Range	Mean	Range
Cervical Spine AP	32	14	18	52.0	20–78	56.9	42–75	1.57	1.42–1.83	23.2	15.9–31.6
Cervical Spine LAT	31	14	17	53.1	20–82	56.4	42–75	1.56	1.42–1.78	23.2	15.9–31.6
Chest PA	110	81	29	55.4	19–87	56.0	38–78	1.60	1.37–1.80	22.0	14.3–35.1
Knee Joint AP	20	9	11	50.4	18–84	56.7	38–78	1.56	1.42–1.73	23.2	16.1–29.0
Knee Joint LAT	21	8	13	50.6	18–84	59.7	40–78	1.55	1.42–1.73	24.7	16.1–29.0
KUB AP	40	26	14	50.1	21–77	57.0	39–74	1.58	1.42–1.73	22.9	15.0–32.8
Lumbar Spine AP	40	17	23	53.7	20–78	56.6	38–76	1.57	1.37–1.73	23.0	14.5–34.8
Lumbar Spine LAT	40	16	24	53.6	18–84	56.0	38–76	1.57	1.37–1.73	22.8	14.5–34.8
Shoulder Joint AP	28	11	17	48.8	22–67	57.9	39–78	1.57	1.40–1.75	23.7	17.2–33.2
Skull PA	20	15	5	44.6	18–74	52.9	39–66	1.61	1.50–1.78	20.4	15.9–26.0
Skull LAT	18	14	4	48.8	19–83	54.3	38–76	1.61	1.47–1.78	20.9	15.9–28.0

Abbreviations: AP, anterior–posterior; KUB, kidney, ureter, and bladder; LAT, lateral; PA, posterior‐anterior.

**FIGURE 1:**
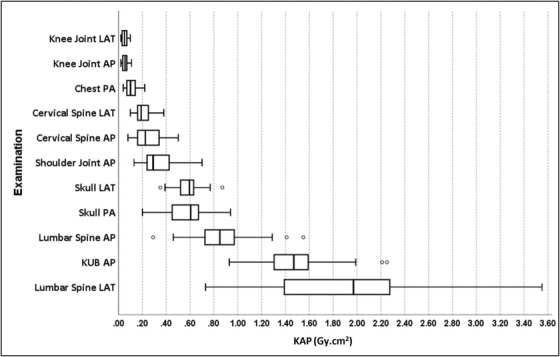
Box plots for kerma‐area product (KAP) values obtained for selected X‐ray examinations. Note: Median values are shown with a vertical line within each box, while the box represents the interquartile range (25th to 75th percentiles). The whiskers represent the distance of 1.5 times the interquartile range (IQR) above the upper quartile (maximum) and below the lower quartile (minimum). AP, anterior‐posterior; LAT, lateral; PA, posterior‐anterior; KUB, kidney, ureter, and bladder

The KAP values calculated for male and female patients for the 11 types of X‐ray examinations are shown in Table 2. It shows the median KAP values as IDRLs and maximum to minimum ratios of KAP for each examination. The IDRLs in Gy.cm^2^ were 0.23 for cervical spine AP, 0.19 for cervical spine LAT, 0.10 for chest PA, 0.06 for knee joint AP, 0.05 for knee joint LAT, 1.47 for KUB AP, 0.85 for lumbar spine AP, 1.97 for lumbar spine LAT, 0.29 for shoulder joint AP, 0.61 for skull PA, and 0.60 for skull LAT examinations. The ratio of maximum to minimum KAP values ranged from 2.4 for KUB AP to 6.3 for cervical spine AP. As shown in Table 2, the lowest mean KAP value was measured for knee joint LAT examination with 0.05 Gy.cm^2^ and the highest for lumbar spine LAT examination with 1.91 Gy.cm.^2^ Figure 1 illustrates box plots for the KAP distribution for each X‐ray examination investigated to better visualize the patient dose distribution. In box plots, the boxes represent the interquartile range (IQR, 25th percentile to 75th percentile) of the distribution, and the whiskers represent the distance of 1.5 times the IQR above the upper quartile (maximum value) and below the lower quartile (minimum value), and the bars inside the box represent the median values. As illustrated in Figure 1, the largest IQR for KAP was observed for the lumbar LAT examination.

**TABLE 2:** The kerma‐area product (KAP) values obtained for male and female patients for selected X‐ray examinations

	KAP (Gy.cm^2^)															
	**Male**					**Female**					**Total**					
Examination type	**Mean (SD)**	**Range**	**25%**	**Median**	**75%**	**Mean (SD)**	**Range**	**25%**	**Median**	**75%**	**Mean (SD)**	**Range**	**25%**	**IDRLs (Median)**	**75%**	**Max/Min Ratio**
Cervical Spine AP	0.26 (0.13)	0.12–0.50	0.16	0.22	0.38	0.24 (0.11)	0.08–0.47	0.16	0.23	0.31	0.25 (0.12)	0.08–0.50	0.16	0.23	0.34	6.3
Cervical Spine LAT	0.22 (0.07)	0.15–0.38	0.16	0.18	0.28	0.20 (0.06)	0.10–0.31	0.14	0.21	0.25	0.21 (0.07)	0.10–0.38	0.16	0.19	0.25	3.8
Chest PA	0.12 (0.05)	0.04–0.22	0.08	0.10	0.15	0.10 (0.04)	0.05–0.17	0.07	0.09	0.13	0.11 (0.04)	0.04–0.22	0.07	0.10	0.14	5.5
Knee joint AP	0.06 (0.02)	0.02–0.10	0.05	0.06	0.08	0.05 (0.03)	0.03–0.11	0.03	0.05	0.07	0.06 (0.03)	0.02–0.11	0.03	0.06	0.07	5.5
Knee joint LAT	0.06 (0.03)	0.02–0.10	0.04	0.06	0.10	0.05 (0.02)	0.03–0.10	0.03	0.04	0.07	0.05 (0.03)	0.02–0.10	0.03	0.05	0.08	5.0
KUB AP	1.44 (0.29)	0.93–1.99	1.23	1.47	1.59	1.53 (0.35)	1.04–2.25	1.33	1.48	1.65	1.47 (0.31)	0.93–2.25	1.30	1.47	1.59	2.4
Lumbar spine AP	0.85 (0.32)	0.29–1.55	0.70	0.79	1.06	0.87 (0.22)	0.46–1.29	0.77	0.87	0.95	0.86 (0.26)	0.29–1.55	0.72	0.85	0.98	5.3
Lumbar spine LAT	2.03 (0.81)	0.73–3.55	1.59	2.06	2.63	1.84 (0.67)	0.73–2.95	1.19	1.94	2.31	1.91 (0.72)	0.73–3.55	1.37	1.97	2.31	4.9
Shoulder joint AP	0.30 (0.10)	0.19–0.47	0.23	0.26	0.42	0.37 (0.16)	0.13–0.70	0.25	0.39	0.52	0.34 (0.14)	0.13–0.70	0.24	0.29	0.43	5.4
Skull PA	0.60 (0.17)	0.31–0.89	0.46	0.61	0.65	0.56 (0.28)	0.20–0.94	0.32	0.54	0.82	0.59 (0.19)	0.20–0.94	0.45	0.61	0.68	4.7
Skull LAT	0.59 (0.12)	0.39–0.77	0.50	0.61	0.65	0.58 (0.22)	0.35–0.87	0.40	0.54	0.79	0.58 (0.14)	0.35–0.87	0.50	0.60	0.65	2.5

Abbreviations: AP, anterior–posterior; KUB, kidney, ureter, and bladder; LAT, lateral; PA, posterior‐anterior; SD, standard deviation; 25%, 25th percentile; 75%, 75th percentile; max/min, maximum to minimum ratio.

Moreover, the descriptive statistics of the radiographic exposure parameters (kVp and mAs) used for each examination type are presented in Table 3. The mean kVp obtained in this study ranged from 53.0 (knee joint AP and LAT) to 106.3 (chest PA). The mean mAs ranged from 3.1 (chest PA) to 58.9 (lumbar spine LAT). The largest kVp range observed for cervical spine AP examination, ranging from 48 to 70. The largest mAs range observed for the lumbar spine LAT examination, ranging from 28.0 to 80.0. Furthermore, the largest IQR for kVp and mAs was observed for chest PA (100–110) and lumbar spine LAT (41.3–71.0) examinations, respectively. The IQR values better express the variability of the kVp and mAs distribution.

**TABLE 3:** Exposure parameters (kVp and mAs) used during selected X‐ray examinations

Examination type	kVp						mAs					
	**Mean**	**SD**	**Median**	**Range**	**25%**	**75%**	**Mean**	**SD**	**Median**	**Range**	**25%**	**75%**
Cervical spine AP	62.7	4.5	60.0	48–70	60.0	66.0	18.5	3.1	18.0	11.0–25.0	16.0	20.0
Cervical spine LAT	65.2	4.1	64.0	60–72	62.0	70.0	19.7	2.6	20.0	12.5–28.0	18.0	20.0
Chest PA	106.3	4.8	110.0	100–110	100.0	110.0	3.1	1.4	2.5	1.3–6.3	2.0	5.0
Knee joint AP	53.0	3.3	53.5	47–58	50.0	55.0	5.8	1.2	5.6	4.0–8.0	5.0	6.9
Knee joint LAT	53.0	3.3	52.0	47–60	50.0	55.0	5.9	1.1	5.6	4.0–8.0	5.0	7.1
KUB AP	67.2	4.6	68.0	58–78	66.0	69.8	35.7	7.4	36.0	20.0–50.0	32.0	40.0
Lumbar spine AP	68.2	4.5	70.0	58–75	66.0	70.0	34.0	8.2	32.0	18.0–56.0	32.0	36.0
Lumbar spine LAT	73.5	3.3	73.0	68–79	70.0	76.0	58.9	16.0	67.0	28.0–80.0	41.3	71.0
Shoulder joint AP	63.8	4.1	63.5	60–73	60.0	66.0	19.2	3.1	20.0	12.5–25.0	18.0	20.0
Skull PA	69.8	1.6	70.0	66–72	70.0	70.0	32.9	6.1	32.0	25.0–50.0	28.0	36.0
Skull LAT	68.1	2.1	68.0	62–70	66.8	70.0	26.7	3.1	25.0	20.0–32.0	25.0	28.0

Abbreviations: AP, anterior‐posterior; KUB, kidney, ureter, and bladder; LAT, lateral; PA, posterior‐anterior; SD, standard deviation; 25%, 25th percentile; 75%, 75th percentile.

Table 4 compares the mean weight, mean exposure parameters (kVp and mAs), and median KAP values obtained in this study with the corresponding values reported from Greece and India.

**TABLE 4:** Comparison of median kerma‐area product (KAP) and mean exposure parameters obtained in the present study with those values reported from India^17^ and Greece.^16^

	Median KAP (Gy.cm^2^)			Mean Weight (kg)			Mean kVp			Mean mAs		
Examination type	**This study**	**India**	**Greece**	**This study**	**India**	**Greece**	**This study**	**India**	**Greece**	**This study**	**India**	**Greece**
Cervical spine AP	0.23	0.21	0.15	56.9	65.0	72.0	63	75	66	18.5	16.4	33.4
Cervical spine LAT	0.19	0.16	0.16	56.4	64.4	72.0	65	75	66	19.7	13.2	27.7
Chest PA	0.10	0.22	0.08	56.0	67.5	71.0	106	120	125	3.1	2.3	2.1
Knee joint AP	0.06	–	–	56.7	–	–	53	–	–	5.8	–	–
Knee joint LAT	0.05	–	–	59.7	–	–	53	–	–	5.9	–	–
KUB AP	1.47	–	1.25	57.0	–	72.0	67	–	81	35.7	–	19.4
Lumbar spine AP	0.85	0.78	0.95	56.6	66.8	72.0	68	70	77	34.0	21.8	35.2
Lumbar spine LAT	1.97	2.72	1.60	56.0	66.4	73.0	74	70	90	58.9	80.1	28.2
Shoulder joint AP	0.29	–	–	57.9	–	–	64	–	–	19.2	–	–
Skull PA	0.61	0.15	–	52.9	63.6	–	70	80	–	32.9	4.1	–
Skull LAT	0.60	0.22	0.27	54.3	64.9	71.0	68	70	77	26.7	4.1	22.3

*Note*: Dash (‐) indicates that the data are not available.

Abbreviations: AP, anterior‐posterior; KUB, kidney, ureter, and bladder; LAT, lateral; PA, posterior‐anterior.

**FIGURE 2:**
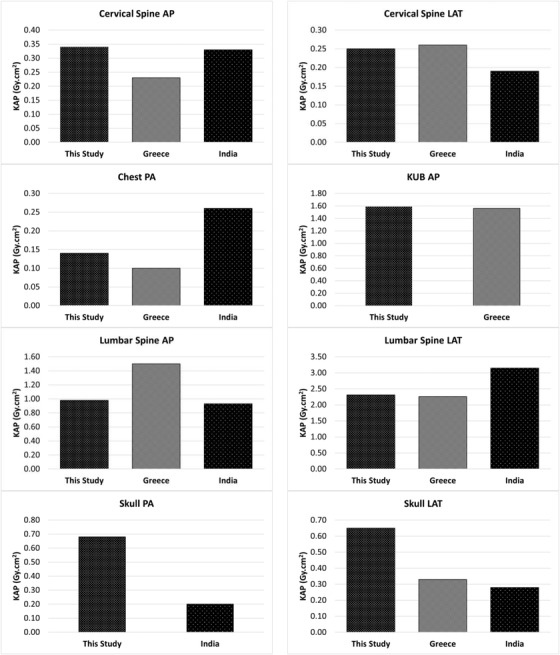
Comparison of 75th percentile in this study with published local diagnostic reference levels (LDRLs) from Greece^16^ and India.^17^ Note: Bars indicate the 75th percentile kerma‐area product (KAP) values (in Gy.cm^2^) resulting from this study and the values reported from Greece and India. The 75th percentiles of the KAP distributions were used to define LDRLs in Greece and India. AP, anterior‐posterior; LAT, lateral; PA, posterior‐anterior; KUB, kidney, ureter, and bladder

In Table 5, the 75th percentile KAP values along with the mean KAP values obtained in this study were compared with the LDRL values reported from Greece and India. The percentage differences were calculated by dividing the differences between two 75th percentile values over the reported LDRL value for a specific examination. The positive increment change was indicated as a positive percentage difference and vice versa. Figure 2 shows the comparison between the 75th percentile KAP values and the LDRL values reported from the Greece and India. There were no published DRLs available for the shoulder joint AP, knee joint AP and LAT examinations.

**TABLE 5:** Comparison of the 75th percentiles (in Gy.cm^2^) in this study with the local diagnostic reference levels (LDRLs) reported from Greece and India

Examination type	This study		Greece^16^		India^17^	
	**Mean**	**75%**	**LDRLs**	**%Difference**	**LDRLs**	**%Difference**
Cervical spine AP	0.25	0.34	0.23	48%	0.33	3%
Cervical spine LAT	0.21	0.25	0.26	−4%	0.19	32%
Chest PA	0.11	0.14	0.10	40%	0.26	‐46%
Knee joint AP	0.06	0.07	–	–	–	–
Knee joint LAT	0.05	0.08	–	–	–	–
KUB AP	1.47	1.59	1.56	2%	–	–
Lumbar spine AP	0.86	0.98	1.50	−35%	0.93	5%
Lumbar spine LAT	1.91	2.31	2.26	2%	3.15	‐27%
Shoulder joint AP	0.34	0.43	–	–	–	–
Skull PA	0.59	0.68	–	–	0.20	240%
Skull LAT	0.58	0.65	0.33	97%	0.28	132%

*Note*: Dash (‐) indicates that the data are not available. The 75th percentiles of the KAP distributions were used to define LDRLs in Greece and India.

Abbreviations: AP, anterior‐posterior; KUB, kidney, ureter, and bladder; LAT, lateral; PA, posterior‐anterior; % Difference, ([75th percentile value in this study – LDRL value]/LDRL value) × 100%.

## 4 DISCUSSION

The present study evaluates the patient doses in terms of KAP in adults who underwent eleven types of projection X‐ray examinations at a public hospital in Uva province. According to ICRP publication 135, the DRL quantity data should be collected for a minimum of 20 patients, including males and females, for a specific examination in a particular institution.^5^ In order to fulfill this recommendation, a sample of a minimum of 20 adult patients was selected for each examination, except for the skull LAT examination. Usually, the weight criterion of 70 ± 10 or 70 ± 20 kg is recommended to standardize patients for small samples of less than 50 patients.^5^ However, in this study, the weight criterion of 58 ± 20 kg was used to standardize the patient sizes according to the patient demographics in Sri Lanka. As shown in Table 1, the mean weight of the present study group was 56.4 kg, which is slightly less than the mean weight of adults in Sri Lanka. Furthermore, the mean weight of this patient group was comparatively lower compared with the Greece (72 kg),^16^ and India (65 kg).^17^ The BMI values were also calculated for each patient using weight and height measurements (weight/(height)^2^) obtained in this study. The BMI range (14.3–35.1 kg m^−2^) of the patients considered in this study is wider than the dosimetric surveys conducted in India (23.9–26.4 kg m^−2^) and Greece (24.8–25.3 kg m^−2^), and it provides a simple and useful classification scheme about the crude size and shape of the patients.^16^, ^17^ The scope of this study was limited to the routinely performed X‐ray examinations in the institution based on their frequencies and contributions to the collective dose in patients.

The obtained KAP values showed wide variations in patient doses for the same type of examination at the same institution, as illustrated in Figure 1. It was observed that the maximum to minimum ratio of KAP values ranged from 2.4 for the KUB AP to 6.3 for the cervical spine AP examinations, indicating the extent of variation. These variations in patient doses require further dose optimization without jeopardizing the diagnostic information. The variations in patient doses could be mainly attributed to several factors, including differences in patient size (weight, height, body thickness), the clinical condition of the patients, exposure parameters (kVp and mAs), techniques employed by radiologic technologists, and equipment conditions. In general, the effect of body size variations on patient doses is minimal for the cervical spine (AP and LAT), and the skull (PA and LAT) examinations due to the small anatomical area exposed compared to other body regions. Therefore, the patient doses in these examinations can easily be standardized. Furthermore, chest PA, KUB AP, lumbar spine AP, and lumbar spine LAT examinations are more complicated to standardize due to the high attenuation of X‐rays and the anatomic region of interest that can vary significantly. In this study, the cervical spine AP examination having the highest ratio of maximum to minimum KAP is considered as abnormal. This may be due to the improper collimation and the selection of exposure parameters. However, the above concerns can often be minimized without compromising image quality or clinical purpose. As shown in Table 2, the patient doses obtained for most X‐ray examinations were comparable among male and female patients, except for lumbar spine LAT examination. For lumbar spine LAT examination, relatively higher patient doses were observed for male patients (2.03 Gy.cm^2^) compared to female patients (1.84 Gy.cm^2^) due to the higher mAs values applied (male patients: 63.1 and female patients: 56.0).

In this study, it was observed that the kVp and mAs were manually adjusted by radiologic technologists using MEC mode. However, the MEC mode is an experience‐related skill, and it has a considerable influence on the patient's dose.^23^ During the study, it was noticed that relatively higher exposure parameters were usually used to obtain a radiograph of patients with larger body sizes but resulted in increased radiation doses. As shown in Table 3, the variation of kVp used in this study was minimal for eleven types of X‐ray examinations, and the SD values also supported it. Large variations in mAs values were observed for lumbar spine LAT (28.0 to 80.0 [SD = 16.0]), lumbar spine AP (18.0 to 56.0 [SD = 8.2]), and KUB AP (20.0 to 50.0 [7.4]) for KUB AP examinations, which also resulting in a wide variation in patient doses for the same examination. The variations in applied kVp and mAs values may be explained by the manual selection of exposure parameters based on patient and/or body regions imaged. The use of high kVp increases the beam penetration, but low mAs result in a reduced dose for the patients.^16^, ^18^ For instance, it was observed that a combination of high kVp (100–110), and low mAs (1.3–6.3) was used for chest PA, which is comparable to the European criterion.^18^ A proper adjustment of exposure parameters respective to patient size is always required for dose optimization. Therefore, high kVp and low mAs techniques are recommended whenever possible to perform X‐ray examinations.

The median KAP and mean weight, kVp, and mAs values used in this study were compared to those reported in India and Greece in Table 4. The reported studies in India and Greece were conducted on a single DR system similar to the present study with 300 and 1504 patients, respectively.^16^, ^17^ The mean kVp values for most of the examinations were lower than the corresponding values for India and Greece. However, the mean mAs values were significantly higher than those used in India, except for the lumbar spine LAT examination. Furthermore, the mAs values reported in Greece were comparable to or lower than the present values, except for cervical spine AP and LAT examinations. The patient group considered in this study was less in mean weight as compared with those in India and Greece for all X‐ray examination types. The median KAP (proposed IDRLs) obtained in Gy.cm^2^ were 0.23 for cervical spine AP, 0.19 for cervical spine LAT, 0.10 for chest PA, 0.06 for knee joint AP, 0.05 for knee joint LAT, 1.47 for KUB AP, 0.85 for lumbar spine AP, 1.97 for lumbar spine LAT, 0.29 for shoulder joint AP, 0.61 for skull PA, and 0.60 for skull LAT examinations. The median KAP values obtained for skull PA and LAT examinations in the present study were significantly higher than in Greece and India. This could be explained by the higher mAs values used for skull PA (8.0 times) and LAT (6.5 times) examinations compared to the corresponding values in India.^17^

The mean and 75th percentiles of the KAP distribution were compared with the LDRLs reported from Greece and India, as presented in Table 5. Dose measurements obtained on DR systems were used to establish the corresponding LDRLs in Greece and India. There was no DRL data available for shoulder joint AP, knee joint AP, and knee joint LAT examinations. In Table 5, our 75th percentile KAP values for skull PA and LAT examinations were respectively 240% and 132% higher than the corresponding LDRLs observed in the study conducted in India.^17^ Moreover, the study conducted in Greece shows that the skull LAT examinations were 97% lower than our 75th percentile KAP value. The significantly higher patient doses are due to the increased mAs values employed in skull PA and LAT examinations. However, it was observed that our 75th percentile KAP value for chest PA examinations was 46% lower than the LDRL value observed in the same study conducted in India.^17^ Overall, the median and the 75th percentile KAP values of most X‐ray examinations were comparable to the corresponding KAP values reported from Greece, and India. Since the median and the 75th percentile KAP values for skull PA and LAT examinations were higher than the reported values, further optimization and a review of dose values are recommended to improve patient protection. For instance, appropriate corrective actions, such as adjusting radiographic exposure parameters (recommend using IQR of kVp and mAs values for patients weighing 38–78 kg) and collimation, can be used to reduce patient doses. Furthermore, the X‐ray system used in this study had a total filtration of 2.5 mm Al, which satisfies the minimum standard filtration requirement of 2.5 mm Al for X‐ray systems operating at above 70 kVp (the minimum standard filtration requirement by the United States Food and Drug Administration is 2.9 mm Al at 81 kVp).^24^ In general, the KAP values obtained for high kVp examinations may be influenced by filtration due to the absorbance of low‐energy radiation by the patient body without contributing to the image quality. Therefore, the use of additional filtration is helpful in reducing patient doses by removing the low‐energy radiation in high kVp settings.^16^

The DRLs are unique and specific to institutions, mainly due to differences in patients’ body dimensions, radiographic techniques used, and equipment modalities. Therefore, DRLs cannot be adopted from other institutions rather than used as a reference guide.^11^ This study emphasizes that each institution should evaluate its own DRLs based on its radiographic techniques and practices to optimize patient doses. The overall findings of this preliminary study ensured that radiologic technologists in this hospital followed the “ALARA” principle to obtain the radiographic images, except for the skull PA and LAT examinations. Therefore, the proposed IDRLs for projection radiography examinations provide a good indication of the current radiology practice in the institution. The radiology staff should be encouraged to minimize dose variations whenever possible to maintain the appropriate level of good practice and performance without compromising diagnostic information. Also, the need for medical physicists in the radiology department was further recognized to optimize patient doses in diagnostic radiographic procedures. A report with recommendations was submitted to the radiology department of the hospital based on the outcome of this study. Special emphasis was given for the need of dose optimization for skull PA and LAT examinations.

## 5 CONCLUSION

The KAP of adult patients underwent cervical spine AP, cervical spine LAT, chest PA, knee joint AP, knee joint LAT, KUB AP, lumbar spine AP, lumbar spine LAT, shoulder joint AP, skull PA, and skull LAT examinations were assessed, and median KAP values obtained were proposed as IDRLs. Most of the median and the 75th percentile KAP values obtained were comparable to corresponding reported KAP values from Greece and India. However, a considerable variation in KAP values and exposure parameters was observed for the skull PA and LAT examinations, indicating the need for optimization of patient doses and exposure parameters in this institution. Hence, the interquartile ranges of exposure parameters are recommended to improve the optimization of patient doses. Overall, this preliminary study can be used as a template for other institutions to establish their own IDRLs.

## AUTHOR CONTRIBUTIONS

Sachith Welarathna designed the study, collected the data, performed the statistical analysis, and drafted the original manuscript. Sivakumar Velautham conceived the study, supervised the study, and edited the manuscript. Sivananthan Sarasanandarajah collaborated on the revision of the manuscript and supervised the study. M. Wanninayake contributed to the initial discussion and funding acquisition. All authors read and approved the final manuscript.

## CONFLICT OF INTEREST

The authors have no conflict of interest to disclose.

## ETHICS STATEMENT

Ethical approval for this study was obtained from the ethical review committee of the National Hospital of Sri Lanka (AAJ/ETH/COM/2022).

## Data Availability

The data that support the findings of this study are available from the corresponding author upon reasonable request
